# Supplementary figures and images for: Ozone ultrafine bubble water exhibits bactericidal activity against pathogenic bacteria in the oral cavity and upper airway and disinfects contaminated healthcare equipment
Source: PLoS One. 2023 Apr 12;18(4):e0284115. doi: 10.1371/journal.pone.0284115 (PMC10096490; doi:10.1371/journal.pone.0284115)

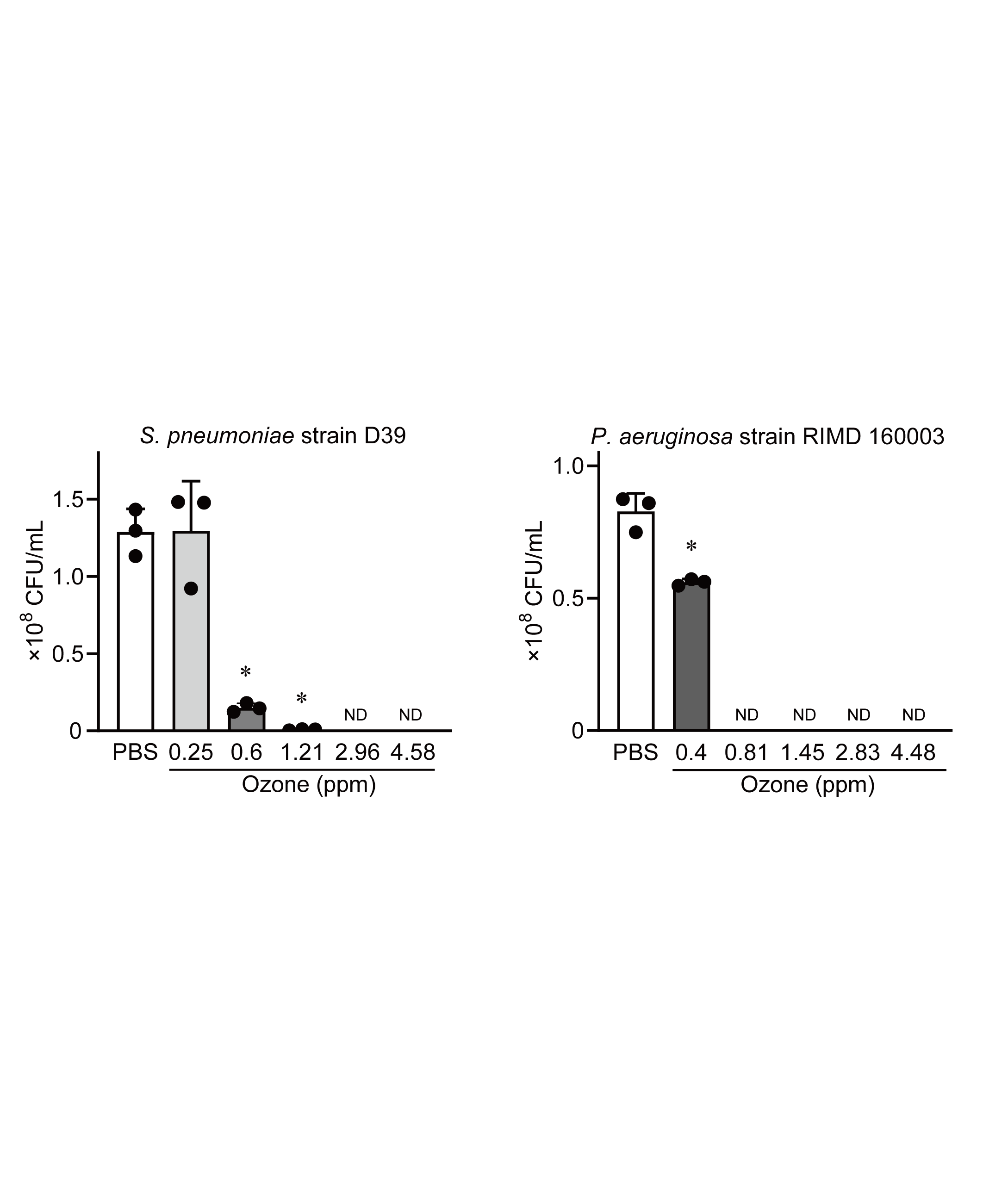

Supplement: S1 Fig — S. pneumoniae strain D39 and P. aeruginosa strain RIMD 1603003 were exposed to 0.4–5 ppm OUFBW for 1 min. Data are presented as the mean ± SD of triplicate experiments and were evaluated using analysis of variance with Dunnett’s multiple-comparisons test. *P < 0.05 compared to the control group. ND stands for undetected and indicates below the detection limit (< 100 CFU/mL). (TIF) [file pone.0284115.s001.tif]
